# Convergent to be divergent: the impact of occupational calling on employees’ creativity

**DOI:** 10.3389/fpsyg.2026.1729967

**Published:** 2026-02-11

**Authors:** Tianyu Liu, Qiong Wu, Lifeng Han

**Affiliations:** 1 School of Business, Macau University of Science and Technology, Macau, China; 2 School of Public Administration, Shandong Normal University, Shandong, China

**Keywords:** ability to focus, employees’ creativity, inclusive leadership, mindfulness, occupational calling

## Abstract

Enhancing employees’ creativity is of great significance for organizations. However, whether, how and when occupational calling enhances employees’ creativity has largely been ignored. To fill this gap, our study draws on mindfulness theory and proposes a dual-stage moderated mediation model by introducing a mediating mechanism of mindfulness and boundary conditions of ability to focus and inclusive leadership. Survey data were collected from 297 respondents using a multi-source time-lagged design. Our research findings revealed a significant positive influence of occupational calling on mindfulness, which in turn positively affects employees’ creativity. Furthermore, ability to focus (as a synergistic enhancer) in the first stage and inclusive leadership (as an effective facilitator) in the second stage were found to amplify these positive effects. All in all, our study provides significant theoretical and practical implications for scholars and practitioners who seek to promote employees’ creativity.

## 1 Introduction

Creativity is the prerequisite for all inventions and creations, forming the foundation for a nation’s enduring vitality. In today’s hyper-competitive 21st-century global landscape, innovation is a defining feature of core competitiveness for nations and organizations alike. From tackling complex public policy issues to creating groundbreaking technologies, creativity has evolved from a prized personal attribute to a vital strategic resource. For organizations, employees’ creativity substantially contributes to the workplace (Anderson et al., 2014) and enhances competitive advantage (Gong et al., 2009), constituting a key component of organizational innovation capability. For individuals, creativity means greater challenges and more opportunities. How to enhance employees’ creativity has thus become a primary concern for managers.

Extensive prior research indicates that employees’ creativity is influenced by both individual differences such as personality, knowledge, skills, abilities, affective and motivational states, and contextual factors such as task characteristics, leadership, relationships with coworkers, as well as organizational culture, structure, policies, and management practices (Zhou and Hoever, 2023, p. 110). For instance, research showed that positive emotions help stimulate employees’ creativity (Amabile et al., 2005), and perceived organizational support can also elicit higher creativity (Kurtessis et al., 2017). Furthermore, employees’ creativity is affected by leadership style. For example, supportive leadership was founded to be more likely to enhance creativity (Lee et al., 2020). Offering rewards or task choices also contributes to increased creativity, particularly when the rewards benefit others (Patall et al., 2008; Zhou et al., 2022). Despite these substantial findings, given that a considerable number of contemporary employees have started planning and developing their professional careers, little research has been conducted to explore the impact of employees’ career construction on their creativity (Savickas, 2005). This research gap manifests practically in the following question: why do employees still lack creativity at work today, despite significant improvements in work environment, leadership approachability, job contents, and rewards compared to the past?

Our study aims to fill this research gap by linking occupational calling—a long-standing concept in sociology that remains underdeveloped in organizational management—to employees’ creativity. Occupational calling refers to a transcendent summons to approach a particular life role in pursuit of meaningfulness that holds altruism as a primary source of motivation (Dik and Duffy, 2009). This definition frames occupational calling as a process integrating personal and social values in career construction (Savickas, 2005). Max Weber, a century ago, used the religiously connotated term “calling” to explain the formation of the capitalist spirit (Weber, 1930). If the early protestants’ sense of calling could generate immense, tireless work motivation, then the secularized concept of occupational calling should likewise enhance contemporary employees’ creativity. Existing research has indeed found that occupational calling can predict employees’ creativity, for instance, research has showed that occupational calling enhances employees’ creativity via career commitment (Lv et al., 2021), and grit (Wang and Su, 2025). However, none of these studies has clearly explained why occupational calling, as a convergent form of thinking (focusing solely on work fulfillment), can promote employees’ creativity, which is a divergent form of thinking.

Grounded in mindfulness theory, our study tries to reconcile this antinomy by investigating the mechanism through which occupational calling enhances employees’ creativity. Specifically, we propose that employees with a strong occupational calling exhibit higher level of mindfulness, defined as a receptive, non-judgmental, and continuous awareness of present-moment state (Brown and Ryan, 2003). The reason for choosing mindfulness as the mediating mechanism is that employees with a high sense of occupational calling focus their attention and energy on work that capitalizes on their talents and is their destiny (Dobrow et al., 2023), and mindfulness precisely captures this state of being fully absorbed in the present-moment. Mindfulness enables employees to fully immerse themselves in their current work, and this state of high work engagement is very conducive to creativity (Langer, 1989).

Additionally, our study examines the boundary conditions of this causal chain. Specifically, we identify two important factors that might influence the process in which employees focus fully on their work to unleash creativity—ability to focus and inclusive leadership. Ability to focus denotes one’s ability to concentrate on productive activities without worrying about having their interests infringed upon by others (Mayer and Gavin, 2005). We posit that higher ability to focus helps employees with a strong occupational calling better eliminate distractions and concentrate on the work itself, acting as a synergistic enhancer for convergent thinking. On the other side, inclusive leadership, characterized by openness, availability, and accessibility, involves soliciting and valuing inputs from employees and recognizing their contributions (Nembhard and Edmondson, 2006). We posit that higher level of inclusive leadership creates a supportive atmosphere where employees can more easily experiment without fear of failure, acting as an effective facilitator for divergent thinking.

Our study contributes to the employees’ creativity literature in at least three ways. First, drawing on mindfulness theory, it explores how occupational calling enhances creativity, extending beyond the predominant focus on the effects of job characteristics, organizational, and leadership factors. Second, it provides a unique explanatory mechanism for the impact of occupational calling on creativity, proposing that occupational calling boosts creativity by enhancing employees’ mindfulness. Finally, it identifies two important boundary conditions, illustrating how ability to focus and inclusive leadership influence the relationships among occupational calling, mindfulness, and creativity.

## 2 Theory and hypotheses

Langer’s mindfulness theory conceptualized mindfulness as an active mode of information processing in which individuals maintain high attention, sharp sensitivity, and non-judgmental acceptance toward their experienced objects (Langer, 1997). Langer’s conceptualization emphasizes active cognitive processing of external stimuli, such as creating new categories, welcoming new information, and seeking more than one view (Langer, 1989). When people act mindfully, it puts them in the present. It makes them more sensitive to context and perspective. It’s this very process of engagement that makes people full of energy (Langer, 2014). Many components of mindfulness are precisely the elements that constitute creativity. If people can break free from stereotypical constraints, remain open to novel and unconventional information, and focus on the process rather than outcomes, they are likely to be innovative persons (Langer, 1989).

After Langer developed the theory of mindfulness, research on this topic has been burgeoning extensively. The scholars’ interest in this concept is partly due to its predictive power with numerous significant outcomes in organizational management research. For instance, previous studies have found that mindfulness could enhance job performance (Dane, 2011), job satisfaction (Andrews et al., 2014), creativity (Baas et al., 2014), and wellbeing (Bajaj et al., 2016); while simultaneously reducing burnout (Hülsheger et al., 2013), and turnover intention (Dane and Brummel, 2014).

### 2.1 Occupational calling and employees’ creativity

Amabile’s (1983) componential model of creativity identifies domain-relevant skills, creativity-relevant processes, and intrinsic task motivation as three essential components for creativity across domains. Domain-relevant skills represent expertise in a specific field, creativity-relevant processes encompass cognitive styles conducive to novel thinking, and intrinsic task motivation refers to individuals’ inherent drive toward their work.

Employees with a strong occupational calling, driven by an internal summons toward meaningful work, often have clearer career choices. They view work as a source of life purpose and meaning, leading to higher occupational passion, work engagement, and career self-efficacy (Dobrow and Tosti-Kharas, 2011; Zhang and Guo, 2023). Since they regard professional achievements as an integral part of their identity, employees with a strong occupational calling continuously explore and deliberately learn in their field, striving for excellence to attain greater professional accomplishments (Bloom et al., 2021; Pratt et al., 2006). As a result, they acquire more domain-relevant skills and become more willing to adopt new perspectives in problem-solving. Furthermore, employees high in occupational calling recognize their work’s importance to the society, exhibiting more altruistic behavior and strengthening intrinsic motivation (Dobrow et al., 2023). The positive relationship between occupational calling and employees’ creativity has also been corroborated by previous research (Duan et al., 2020; Liu and Xu, 2022).

In summary, employees with a strong occupational calling have clearer purpose and more enjoyable work experiences, which in turn enhance creativity. Therefore, we hypothesize:

*H1:* Occupational calling is positively related to employees’ creativity.

### 2.2 The mediating role of mindfulness

Mindfulness refers to the conscious awareness of present experience without judgment (Brown and Ryan, 2003). Mindfulness theory posits that mindfulness is an active cognitive operation characterized by the creation of new categories, the assimilation of new information, and the seeking of multiple perspectives (Langer, 1989). Employees with a strong sense of occupational calling derive fulfillment through engagement with the work, they do not view their work merely as a means of livelihood nor are they solely driven by upward mobility within the occupational hierarchy (Wrzesniewski et al., 1997). Instead, they focus less on immediate gains and more on long-term development, actively attending to new information and feedback from the work itself, thereby enhancing their present-moment awareness (Lysova et al., 2019).

On the other side, mindfulness is associated with an enhanced ability to shift perspectives (Reb et al., 2020). It focuses awareness on the present, freeing it from discriminatory, absolute, and habitual mindset, leading to clearer and fresher awareness that allows for more flexible and objective understanding of mental and behavioral responses (Brown et al., 2007; London et al., 2023). Mindful individuals avoid falling into unconscious, automatic processing traps that lead to rigid behavioral patterns (Langer, 1989). Furthermore, mindfulness creates rather than depletes energy. By maintaining mindfulness and attending to surroundings instead of living mindlessly, individuals can reduce stress (Vonderlin et al., 2020) and unleash creativity (Langer, 2014; Khoury et al., 2015). Therefore, to generate new ideas and solutions, break cognitive fixedness, and gain genuine insight, employees need to avoid premature cognitive commitments, drawing instead on novel perceptual inputs from the present to construct new knowledge. As employees become more mindful, they continuously discover new aspects of their work, keeping the mind open and active, and are more willing to try new methods and accept challenges. Previous research on mindfulness and creativity indicates that mindfulness improves concentration (Sedlmeier et al., 2012), enhances open-mindedness, and reduces fear and self-criticism (Brown et al., 2007).

In summary, employees high in occupational calling exhibit greater mindfulness. Unleashing intuition and transcending context open up new possibilities, thereby stimulating employees’ creativity. Therefore, we hypothesize:

*H2*: Mindfulness mediates the positive relationship between occupational calling and employees’ creativity.

### 2.3 The moderating role of ability to focus

Ability to focus describes the psychological safety employees feel in a specific work environment, enabling them to direct more attention to work activities beneficial to the organization (George and Brief, 1996). This concept derives from research on trust and performance, suggesting that individuals operate within specific social contexts where their cognitions and actions are influenced by environmental factors such as rules and climate, that can constrain the scope for autonomous will (Mayer and Gavin, 2005). From a cognitive resource allocation perspective, individuals have limited cognitive resources. When employees have concerns, they must allocate more attentional resources to identify non-productive information for self-protection (Shoss et al., 2023), resulting in low ability to focus. Conversely, fewer work-irrelevant distractions allow more cognitive resources to be invested in work-related processes, leading to high ability to focus.

Experiencing an occupational calling unifies self and career narratives, and gives personal meaning to work (Bloom et al., 2021), thus making it easier to generate mindfulness toward work. However, previous research suggests that while occupational calling can foster open-mindedness, it may also render individuals more sensitive to negative information (Andel et al., 2021). According to mindfulness theory, the non-judgmental awareness in a mindful state might further increase the allocation of attentional resources to non-work contents (Langer, 1989). This makes the linkage between occupational calling and mindfulness susceptible to interference from work-irrelevant information. Once employees are better able to concentrate on productive activities, they will notice new things, thereby gaining insight into previously unnoticed aspects of the subject matter (Langer, 1997).

In summary, if high calling employees are free from non-productive activities, they can devote all their cognitive resources to extend information and explore new possibilities. Therefore, we hypothesize:

*H3*: Ability to focus moderates the relationship between occupational calling and mindfulness, such that the positive effect of occupational calling on mindfulness is stronger when ability to focus is higher.

### 2.4 The moderating role of inclusive leadership

Employees’ creativity requires supportive organizational work environments (Volery and Tarabashkina, 2021). Previous research has established leadership as a key component of the work environment and a critical factor in organizational change. Supportive, non-controlling leadership styles help create environments conducive to employees’ creativity (Ye et al., 2025). Tan et al. (2021) found that positive leader-member exchange enhances employees’ creativity. When leaders demonstrate support for new ideas, employees perceive it as organizational encouragement for innovation (Musenze and Mayende, 2023; Ye et al., 2022).

Inclusive leadership is regarded as a typical supportive leadership style because leaders value employees’ contributions (Nembhard and Edmondson, 2006). According to mindfulness theory, the most effective leadership practices for interacting with mindful individuals are: emphasizing deep listening to understand others, integrating multiple perspectives to build consensus, while striving to eliminate fear and defensive behaviors that arise during intellectual stimulation processes (Langer, 2014). Inclusive leaders involve employees in discussions and decision-making processes, enabling them to openly discuss, promote, and implement new ideas (Haq et al., 2024). Furthermore, inclusive leadership emphasizes collaboration, demonstrating availability (Shore and Chung, 2022), and encouraging employees to generate and use new ideas (Carmeli et al., 2010). Additionally, inclusive leaders support bold innovation and assume responsibility for failures, allowing employees to pursue innovative endeavors without fear.

In summary, inclusive leaders create a supportive, innovation-encouraging atmosphere in which employees with greater mindfulness can better embrace the new methods and accept challenges. Therefore, we hypothesize:

*H4*: Inclusive leadership moderates the relationship between mindfulness and employees’ creativity, such that the positive effect of mindfulness on employees’ creativity is stronger when the level of inclusive leadership is higher.

Combining Hypothesis 2, 3, and 4, we argue that the indirect effect of occupational calling on employees’ creativity via mindfulness is moderated by ability to focus and inclusive leadership, such that this indirect effect will be more pronounced at higher levels of ability to focus and inclusive leadership. Therefore, we hypothesize:

*H5*: Ability to focus and inclusive leadership moderate the indirect effect of occupational calling on employees’ creativity via mindfulness, such that this indirect effect is stronger when ability to focus and inclusive leadership are higher.

**FIGURE 1:**
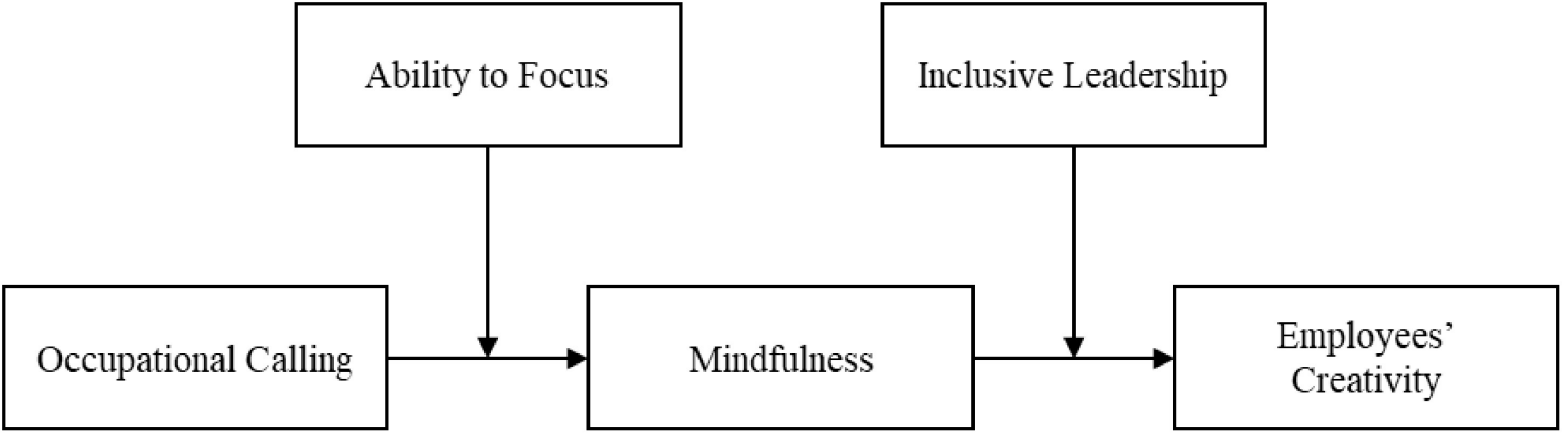
The hypothesized model.

The hypothesized model is presented in Figure 1.

## 3 Materials and methods

### 3.1 Sample and procedure

We collected data via questionnaire surveys from different types of organizations all over China, operating in a wide range of professions including but not limited to doctors, nurses, teachers, and lawyers which are generally considered to have a strong sense of occupational calling.

Surveys were conducted in three waves and a matched employee-leader questionnaire was used. We randomly emailed HR executives from eligible organizations to inquire about their willingness to serve as our liaisons. We then contacted those who responded positively to invite employees and their direct leaders participating in our study, and all participants received an announcement including the research purpose and procedures. A total of 364 employee-leader dyads agreed to take part in. At Time 1 (T1), we measured occupational calling, ability to focus, and control variables, self-reported by employees. Three weeks later at Time 2 (T2), we measured mindfulness and inclusive leadership, also self-reported by employees. Then another three weeks later at Time 3 (T3), we measured employees’ creativity, reported by their direct leaders. No participating employees shared the same direct leader to avoid cross-level nesting issues.

The final sample consisted of 297 employee-leader dyads, yielding a response rate of 81.6%. For the employees, 43.1% were male, 89.2% had a university degree or above, and the mean age was 29.8 years.

### 3.2 Measures

We translated the measures from original English version to Chinese using the translation/back-translation procedure (Brislin, 1980). Participants responded to all items on a 5-point Likert scale (from 1 = *strongly disagree* to 5 = *strongly agree*).

### 3.2.1 Occupational calling

Occupational calling was measured using the Calling and Vocation Questionnaire (CVQ) by Dik et al. (2012). CVQ has 12 items across three dimensions: transcendent summons, purposeful work, and prosocial orientation. A sample item of transcendent summons is: “*I was drawn by something beyond myself to pursue my current line of work.*”; A sample item of purposeful work is: “*My career is an important part of my life’s meaning.*”; A sample item of prosocial orientation is: “*Making a difference for others is the primary motivation in my career*” (α = 0.88).

### 3.2.2 Mindfulness

Mindfulness was measured using the 9-item scale by Haigh et al. (2011), adapted from Bodner and Langer’s (2001) Mindfulness/Mindlessness Scale (MMS). A sample item is: “*I like to investigate things*” (α = 0.84).

### 3.2.3 Ability to focus

Ability to focus was measured using the 6-item scale by Mayer and Gavin (2005). A sample item is: “*The work climate here allows me to focus on doing my job*” (α = 0.83).

### 3.2.4 Inclusive leadership

Inclusive leadership was measured using the Inclusive Leadership Scale (ILS) by Hollander (2012). ILS has 16 items across three dimensions: support-recognition, communication-action-fairness, and self-interest-disrespect. A sample item of support-recognition is: “*My leader asks for my ideas about my work.*”; A sample item of communication-action-fairness is: “*My leader provides clear goals to be achieved.*”; A sample item of self-interest-disrespect is: “*My leader makes comments to put me down (reverse-coded)*” (α = 0.93).

### 3.2.5 Employees’ creativity

Employees’ creativity was measured using the 4-item scale by Farmer et al. (2003). A sample item is: “*This employee generates ground-breaking ideas related to the field*” (α = 0.78).

### 3.2.6 Control variables

We controlled for employees’ gender, age, and educational level—demographic variables considered to affect employees’ creativity (Baer et al., 2021).

### 3.3 Analytic strategy

Mediation, moderation, and moderated mediation analyses were conducted using the PROCESS macro for SPSS to generate the 95% bias-corrected bootstrapped confidence intervals (CI) for the indirect and conditional indirect effects (Hayes, 2013). PROCESS Model 4 was adopted for mediation, and PROCESS Model 21 was adopted for moderation and moderated mediation.

## 4 Results

We first conducted a confirmatory factor analysis to examine the discriminant validity of the constructs. Given the relatively large number of items in two measures—occupational calling and inclusive leadership, we applied item parceling technique to address the biased parameter estimates associated with large number of items relative to the sample size (Little et al., 2002). Specifically, we used *Factorial* algorithm to assign indicators to parcels, with each parcel sequentially assigned the indicators with the highest and lowest factor loadings in alternating direction (Rogers and Schmitt, 2004). Results showed that our hypothesized 5-factor model had good fit (χ^2^/*df* = 2.19, CFI = 0.92, TLI = 0.91, RMSEA = 0.06) and fit better than several alternative models (see Table 1), indicating that our 5-factor model best describes the data.

**TABLE 1:** The results of confirmatory factor analysis.

Model	χ^2^	*df*	χ^2^/*df*	CFI	TLI	RMSEA	Δχ^2^
Hypothesized 5-factor model	579.88	265	2.19	0.92	0.91	0.06	Δχ^2^(4) = 180.63***
4-factor model (combine OC and CR)	760.51	269	2.83	0.87	0.86	0.08	
4-factor model (combine MF and CR)	717.40	269	2.67	0.88	0.87	0.08	Δχ^2^(4) = 137.52***
4-factor model (combine MF and IL)	959.24	269	3.57	0.82	0.80	0.09	Δχ^2^(4) = 379.36***
4-factor model (combine AF and IL)	794.53	269	2.95	0.86	0.85	0.08	Δχ^2^(4) = 214.65***

*n* = 297. OC, occupational calling; CR, employees’ creativity; MF, mindfulness; AF = ability to focus; IL = inclusive leadership.

***p < 0.001.

Although we obtained data from two different sources in three waves, our study may still be subject to the potential common method bias problem. Therefore, we employed Harman’s single-factor test to check whether there exists a single factor that can explain most of the covariance among the measures. The results showed that the first factor had an eigenvalue of 14.91, only accounting for 31.73% of the covariance, which is below the critical value of 40%, suggesting that common method bias was not a serious problem in our study (Podsakoff et al., 2003).

The means, standard deviations, and correlations for all variables are presented in Table 2. The results showed that occupational calling was positively related to mindfulness (*r* = 0.52, *p* < 0.001) and employees’ creativity (*r* = 0.55, *p* < 0.001), and mindfulness was positively related to employees’ creativity (*r* = 0.55, *p* < 0.001), thus providing initial support for our hypotheses.

**TABLE 2:** Means, standard deviations, and correlations for all variables.

Variable	Mean	SD	1	2	3	4	5	6	7	8
1. Gender^a^	1.57	0.50	–0.01	–0.06		(0.88)	(0.84)	(0.83)	(0.93)	(0.78)
2. Age	29.80	8.04								
3. Education^b^	3.03	0.62	0.12*							
4. Occupational calling	3.96	0.61	–0.15**	0.04	0.04					
5. Mindfulness	4.12	0.56	–0.16**	0.04	0.04	0.52***				
6. Ability to focus	3.66	0.80	–0.06	0.11	0.11	0.38***	0.43***			
7. Inclusive leadership	4.03	0.64	–0.06	0.06	0.09	0.53***	0.54***	0.63***		
8. Employees’ creativity	4.00	0.66	–0.16**	0.02	0.04	0.55***	0.55***	0.35***	0.53***	

*n* = 297. Cronbach’s alphas are in parentheses on the diagonal.

^a^1 = male, 2 = female.

^b^1 = high school and below, 2 = college, 3 = university, 4 = master degree and above.

*p < 0.05;

**p < 0.01;

***p < 0.001.

Tables 3, 4 summarize the results of our hypotheses testing. Hypotheses 1 proposed that occupational calling is positively related to employees’ creativity. Consistent with our hypothesis, after controlling for demographics, occupational calling was positively related to employees’ creativity (*b* = 0.58, *p* < 0.001; see Table 3). Therefore, Hypotheses 1 was supported.

**TABLE 3:** The Results of the path analysis.

Model	Total effect	Standard error	LLCI	ULCI
OC→CR	0.58***	0.05	0.48	0.69
	Direct effect	Standard error	LLCI	ULCI
OC→CR	0.39***	0.06	0.28	0.51
	Indirect effect	BootSE	BootLLCI	BootULCI
OC→MF →CR	0.19	0.06	0.09	0.34

*n* = 297. Unstandardized estimates are reported. OC, occupational calling; CR, employees’ creativity; MF, mindfulness.

***p < 0.001.

Hypotheses 2 proposed that mindfulness mediates the positive relationship between occupational calling and employees’ creativity. Results based on 5,000 bootstrap samples showed that after controlling for demographics, the indirect effect confidence interval excluded zero (effect = 0.19, 95% CI = [0.09, 0.34]; see Table 3). Therefore, Hypotheses 2 was supported.

**FIGURE 2:**
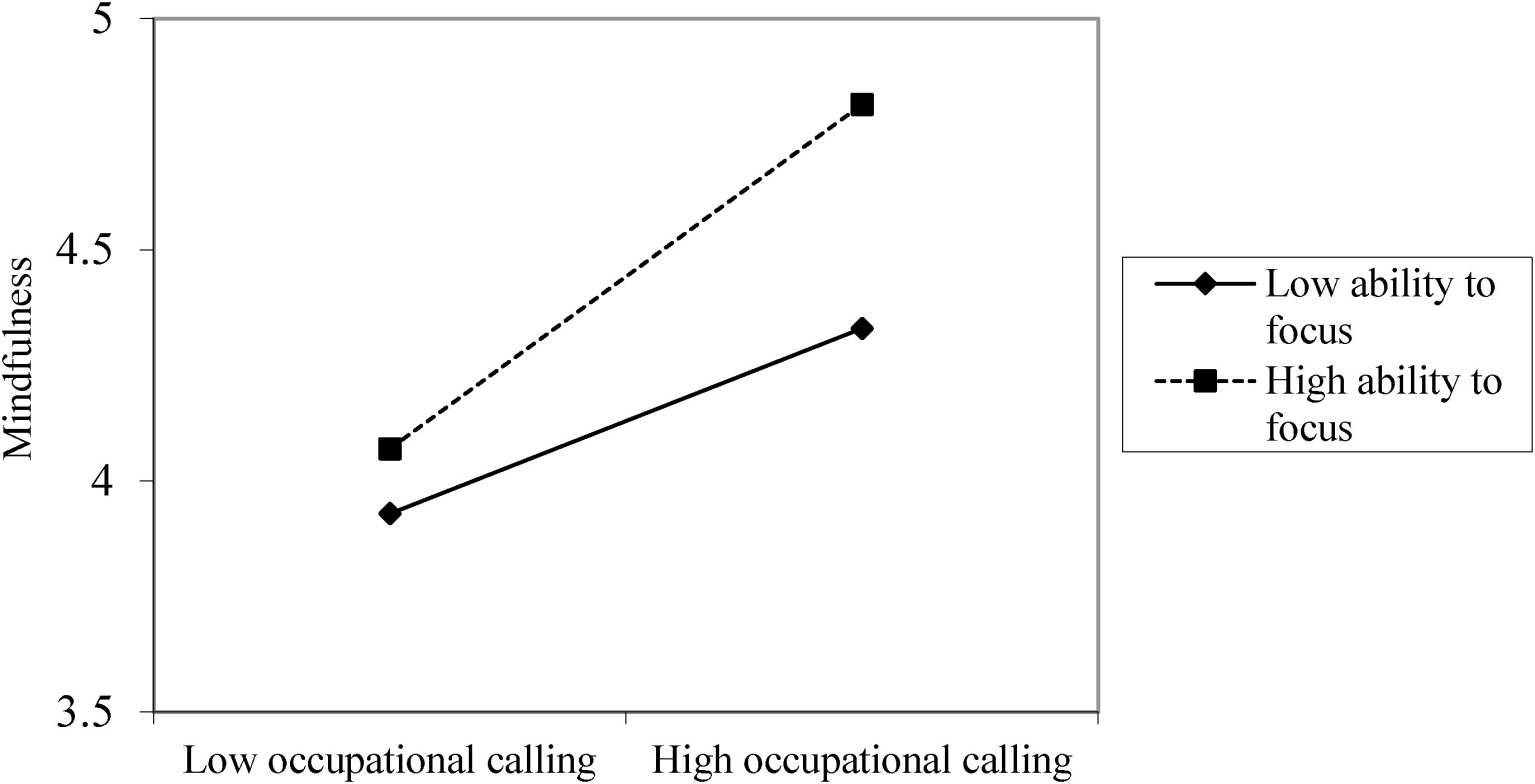
The interactive effect of occupational calling and ability to focus on mindfulness.

Hypotheses 3 proposed that ability to focus moderates the relationship between occupational calling and mindfulness. Consistent with our hypothesis, after controlling for demographics, the interaction term of occupational calling and ability to focus was positively related to mindfulness (*b* = 0.18, *p* < 0.001; see Table 4). A simple slopes analysis further showed that occupational calling was more positively related to mindfulness at higher level of ability to focus (*b* = 0.61, *p* < 0.001) than at lower level of ability to focus (*b* = 0.33, *p* < 0.001; difference = 0.28, *p* < 0.001) (see Figure 2). Therefore, Hypothesis 3 was supported.

**TABLE 4:** The results of the moderation.

Predictor	Mindfulness	Employees’ creativity
Intercept	0.17 (0.18)	4.09*** (0.19)
Gender	–0.10 (0.05)	–0.09 (0.06)
Age	–0.002 (0.003)	–0.004 (0.004)
Education	0.003 (0.04)	0.04 (0.05)
Occupational calling	0.47*** (0.05)	0.27*** (0.06)
Mindfulness		0.54*** (0.08)
Ability to focus	0.20*** (0.04)	
Inclusive leadership		0.24*** (0.06)
Occupational calling × ability to focus	0.18*** (0.04)	
Mindfulness × inclusive leadership	0.38	0.33*** (0.07)
*R* ^2^		0.48
*F*	29.20***	38.23***

*n* = 297. Unstandardized estimates are reported. Standard errors are in parentheses. All variables that define products are mean centered.

***p < 0.001.

**FIGURE 3:**
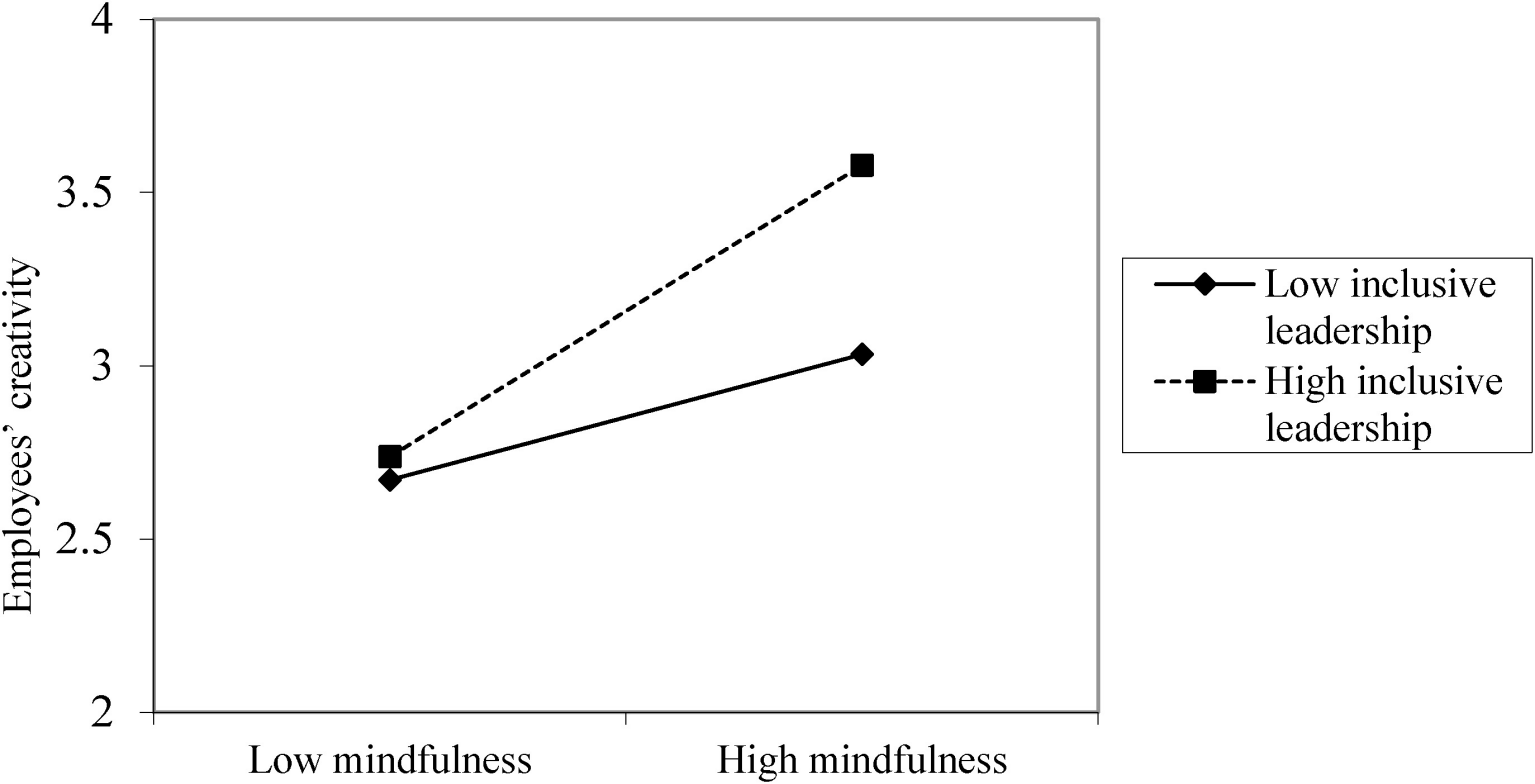
The interactive effect of mindfulness and inclusive leadership on employees’ creativity.

Hypotheses 4 proposed that inclusive leadership moderates the relationship between mindfulness and employees’ creativity. Consistent with our hypothesis, after controlling for demographics, the interaction term of mindfulness and inclusive leadership was positively related to employees’ creativity (*b* = 0.33, *p* < 0.001; see Table 4). A simple slopes analysis further showed that mindfulness was more positively related to employees’ creativity at higher level of inclusive leadership (*b* = 0.75, *p* < 0.001) than at lower level of inclusive leadership (*b* = 0.33, *p* < 0.001; difference = 0.43, *p* < 0.001) (see Figure 3). Therefore, Hypothesis 4 was supported.

Hypotheses 5 proposed that ability to focus and inclusive leadership moderate the indirect effect of occupational calling on employees’ creativity via mindfulness. Results based on 5,000 bootstrap samples showed that after controlling for demographics, the moderated mediation CI excluded zero (index = 0.06, 95% CI = [0.001, 0.13]). Specifically, occupational calling had a more positive indirect effect on employees’ creativity at higher level of ability to focus and inclusive leadership (effect = 0.46, 95% CI = [0.22, 0.74]), than at lower level of ability to focus and inclusive leadership (effect = 0.11, 95% CI = [0.02, 0.24]). Moreover, there was a significant difference in indirect effects between the two conditions (contrast effect = 0.35, 95% CI = [0.12, 0.61]). A closer examination revealed that occupational calling had more positive indirect effects on employees’ creativity at higher level of ability to focus and inclusive leadership than at higher level of ability to focus and lower level of inclusive leadership (effect = 0.20, 95% CI = [0.03, 0.42]; contrast effect = 0.26, 95% CI = [0.02, 0.48]), and at lower level of ability to focus and higher level of inclusive leadership (effect = 0.25, 95% CI = [0.11, 0.42]; contrast effect = 0.21, 95% CI = [0.04, 0.43]). Therefore, Hypothesis 5 was supported.

## 5 Discussion

Our study investigated the antecedents of employees’ creativity from a novel perspective of occupational calling, and boundary conditions affecting this relationship. The results revealed some important theoretical implications. First, occupational calling positively influences creativity. Employees with a strong occupational calling exhibit higher intrinsic motivation, work meaningfulness, and prosocial motivation. Throughout their long careers, to continuously meet their professional needs and achieve self-fulfillment and personal accomplishments, they actively acquire knowledge in relevant fields, constantly adapt their ways of thinking, and stimulate new reflections, thereby elevating their level of creativity (Bacouël et al., 2024).

Second, based on mindfulness theory, our study examined for the first time the mediating mechanism of mindfulness in the relationship between occupational calling and employees’ creativity. Mindfulness promotes conscious present-moment awareness, preventing absolute, automatic, and rigid behavioral patterns and fostering flexible perception of situations. This helps employees notice new aspects of work, keeping the mind open and receptive to new methods and challenges. On the other hand, when they notice new things, they start to see previously unseen aspects, and things look different when approaching from different perspectives. This kind of open-mindedness can foster creativity (Op den Kamp et al., 2023; Petrou et al., 2024). In so doing our study points to a fascinating antinomy: you can only demonstrate divergent thinking by first exhibiting convergent thinking.

Finally, ability to focus moderates the effect of occupational calling on mindfulness, while inclusive leadership moderates the effect of mindfulness on employee’ creativity. When employees are free from concerns about non-productive information, those with a strong occupational calling can better focus their cognitive resources on the work itself. This enables them to pay more attention to new information in their tasks and increases the likelihood of approaching problems from multiple perspectives (Unsworth et al., 2024). On the other side, a supportive organizational climate allows highly engaged employees to better utilize their talents. Inclusive leadership, through its open, available, and accessible nature, fosters a positive atmosphere that encourages experimentation without fear of failure. Positive feedback from leaders enhances perceived control and enables adaptation for better outcomes. By supporting employees and aligning personal and organizational goals, inclusive leadership motivates deeply engaged employees to realize their self-potential and unleash greater creativity (Ly, 2024).

### 5.1 Practical implications

Our study has several important practical implications for managers who want to improve employees’ creativity. First, managers may recruit employees with a strong occupational calling for positions requiring creativity. Simultaneously, they should provide meaning-based training for existing employees to help them discover their calling. Managers also need to encourage and assist employees in setting meaningful work goals aligned with organizational objectives, giving them autonomy to find effective ways to achieve them.

Second, as the results show that employees’ mindfulness serves as a pivotal role in evoking creativity, managers should keep track of their employees’ mindfulness at work by encouraging them to broaden their perspectives, mindfully notice new things, and avoid falling into the trap of old thinking patterns. More important, managers need to create a psychologically safe atmosphere for employees, shielding them from non-productive distractions so they can focus solely on the work itself, consciously developing mindful thinking in pursuit of excellence.

Finally, organizations can provide leadership-based training for managers to help them develop an inclusive leadership style and foster a supportive organizational atmosphere. Managers should act as supporters, putting employees first, granting employees sufficient autonomy, and collaborating with them around mutually agreed-upon goals. Throughout this process, managers need to set clear goals and continuously provide feedback and guidance on challenges employees encounter, encouraging them to take initiative in experimentation and fully leverage their creativity.

### 5.2 Limitations and future directions

Our study has inevitably some limitations. First, we explored the impact of occupational calling on employees’ creativity, primarily based on the mindful state generated by employees in the workplace. However, the emergence of employees’ creativity is an extremely complex process in which emotions also play a significant role. While our study focuses on the cognitive patterns that stimulate creativity, specific emotional experiences of employees (e.g., flow) may also be important mechanisms that link occupational calling and employees’ creativity. Furthermore, previous research has identified motivational factors like intrinsic motivation, creative self-efficacy, and prosocial motivation, as the significant antecedents of employees’ creativity (Liu et al., 2016). Motivational mechanisms, along with cognitive and emotional mechanisms, independently and interactively play critical roles between the effect of occupational calling on employees’ creativity.

Second, we only considered ability to focus and inclusive leadership as boundary conditions affecting the relationship between occupational calling and employees’ creativity. In fact, other factors may also influence this relationship. For instance, different institutional designs, organizational climates, or employee relationships could potentially enhance or diminish employees’ focus at work, thereby impacting their creativity.

Third, regarding the conception and measurement of occupational calling, which originates from Western religious cultural contexts, it is not yet clear whether it aligns with the deep identification of work among Chinese employees.

Finally, an extension to our study can be conducted in several ways. First, future research should examine other boundary conditions under which occupational calling influences employees’ creativity, particularly the inhibitory factors. Exploring these negative factors can help organizations eliminate potential risks in advance. Second, it will be worthwhile for future research to investigate whether there is a one-to-one correspondence between the three dimensions of occupational calling, the three characteristics of mindfulness, and the three components of creativity. Third, team-level phenomena deserve more attention. An important theoretical question for future research is how the overall level and diversity of employees’ occupational calling within a team affect collective mindfulness and team creativity.

## 6 Conclusion

Our study explores how occupational calling enhances employees’ creativity. The results indicate that occupational calling improves employees’ creativity by enhancing their mindfulness. Ability to focus and inclusive leadership jointly strengthen this mechanism. The findings provide important theoretical and practical implications for helping employees exhibit mindfulness and stimulate creativity.

## Data Availability

The original contributions presented in this study are included in this article/supplementary material, further inquiries can be directed to the corresponding author.
